# Knowledge, perceived stigma, and care-seeking experiences for sexually transmitted infections: a qualitative study from the perspective of public clinic attendees in Rio de Janeiro, Brazil

**DOI:** 10.1186/1471-2458-7-18

**Published:** 2007-02-01

**Authors:** Monica Malta, Francisco I Bastos, Steffanie A Strathdee, Shayna D Cunnigham, Jose Henrique Pilotto, Deanna Kerrigan

**Affiliations:** 1 Social Science Department, Sergio Arouca School of Public Health (DCS/ENSP), Oswaldo Cruz Foundation, Rio de Janeiro, Brazil; 2 Mental Health Department, Bloomberg School of Public Health, Johns Hopkins University, Baltimore, MD, USA; 3 Health Information Department, Center for Scientific and Technological Information (DIS/CICT), Oswaldo Cruz Foundation, Rio de Janeiro, Brazil; 4 Division of International Health and Cross Cultural Medicine, Department of Family and Preventive Medicine at University of California, San Diego (UCSD) School of Medicine, San Diego, CA, USA; 5 Department of International Health, Bloomberg School of Public Health, Johns Hopkins University, Baltimore, MD, USA; 6 Evandro Chagas Clinical Research Institute, Oswaldo Cruz Foundation, Rio de Janeiro, Brazil

## Abstract

**Background:**

An estimated 12 million sexually transmitted infections (STIs) are documented in Brazil per year. Given the scope of this public health challenge and the importance of prompt treatment and follow-up counseling to reduce future STI/HIV-related risk behavior, we sought to qualitatively explore STI clinic experiences among individuals diagnosed with STIs via public clinics in Rio de Janeiro, Brazil. The study focused on eliciting the perspective of clinic users with regard to those factors influencing their STI care-seeking decisions and the health education and counseling which they received during their clinic visit.

**Methods:**

Thirty semi-structured interviews were conducted with heterosexual men and women and men who have sex with men presenting with STIs at two public clinics. Content analysis was conducted by coding transcripts of audio-taped interviews for key domains of interest and comparing and synthesizing code output across participants and sub-groups. Thematic narratives were then developed per each of the study sub-groups.

**Results:**

Salient themes that emerged from participant narratives included the importance of low STI-related knowledge and high perceived stigma, both STI-related and other types of social stigma, on STI care-seeking delays. However, there are indications in the data that the level of STI-related knowledge and the amount and types of stigma experienced vary across the study sub-groups suggesting the need for further research on the significance and program relevance of these potential differences. Interview findings also suggest that such barriers to care seeking are not adequately addressed through ongoing health education and counseling efforts at public STI clinics and in turn critical opportunities for STI/HIV prevention are currently being missed.

**Conclusion:**

Information, communication and education regarding early recognition and prompt care-seeking for STIs should be developed, with consideration given to the possibility of tailoring messages tailored to specific sub-groups. To promote prompt treatment-seeking, interventions must also address both STI-specific and other forms of social stigma which may limit access to care. Efforts to further assess and respond to barriers related to the delivery of quality health education and counseling within the context of public STI clinics are also needed.

## Background

Sexually transmitted infections (STIs) account for a major burden of disease in many developing countries. In Brazil, an estimated 12 million people are infected with an STI per year [1]. Left untreated, STIs can result in pelvic inflammatory disease, infertility, various cancers as well as adverse pregnancy outcomes [2]. Beyond being a major public health problem in their own right, both viral and bacterial STIs increase susceptibility to and transmission of HIV [3]. Identifying barriers to potentially infected persons seeking STI-related care and ensuring proper treatment of those who test positive is thus of great clinical and public health importance.

One barrier to effective STI control is the high prevalence of asymptomatic disease; however, even persons who experience symptoms often do not seek or delay seeking appropriate diagnostic and treatment services. Several factors may be associated with STI-related care-seeking including: patient characteristics (e.g., gender, age, and education level); type of disease or symptoms; aspects of the health service (e.g., accessibility, cost and quality of care); and features of the cultural setting (e.g., conceptions of health and illness and gender roles and norms) [4]. Among these, two of the most universally acknowledged issues are STI-related knowledge and stigma.

Although possessing information to be able to recognize and correctly interpret one's signs and symptoms as manifestations of an STI is necessary for prompt care-seeking, a lack of knowledge does not always explain delays in care-seeking behavior [5,6]. For example, in a study of adolescents attending a public STI clinic in Chicago, greater levels of knowledge were associated with delayed care-seeking among females [7]. It is possible that fear of stigma or the emotional consequences of having an STI may supercede a person's concerns for their well-being when he/she suspects that they may be infected [8]. STI-related stigma has been associated with delayed care-seeking in studies from several countries including the United States, Great Britain, Netherlands, Vietnam, and Kenya [7,9-12].

In addition to understanding what impedes or facilitates persons with or at-risk for STIs to access clinical care, it is also of public health importance to provide quality health education and counseling to those who do access care in order to prevent new infections. However, research has shown that the opportunity for post-diagnosis STI-related prevention interventions is often missed. In the Brazilian context, for example, post-test STI counseling does not appear to be universally offered or of consistently high quality based on research conducted to date. A study conducted by Giffin and Lowndes (1999) found that of 42 Brazilian women who were interviewed immediately after having received a positive result for Chlamydia in the context of routine gynecological care, only two understood that the disease was sexually transmitted and only four knew the correct name of the disease. Approximately half of the gynecologists interviewed in this study reported that they did not explicitly inform the patient that her condition was sexually transmitted. The primary reasons for not discussing the specifics of the STI diagnosis cited by providers were the desire to avoid causing potential relationship problems for the couple, particularly in the case that the patient was married, and feeling that dialogue regarding sex and sexuality were beyond the scope of their professional responsibilities [13].

Such findings indicate that there is a further need to explore and improve the quality of post-diagnosis counseling regarding HIV/STI in the Brazilian context. In turn, we employed qualitative research methods to better understand both the factors that influence STI care-seeking and the quality of health education and counseling received among heterosexual women, heterosexual men, and men who have sex with men (MSM) attending two large, public STI clinics in Rio de Janeiro, Brazil. To our knowledge, this is the first study to qualitatively explore STI care-seeking decisions and potential missed opportunities for socio-behavioral interventions in a South American context.

## Methods

Thirty in-depth interviews were conducted with individuals seeking care at two large public STI clinics located in the greater Rio de Janeiro area during the period 2002–2003. Such public clinics generally serve lower-income individuals in the Brazilian context where a system of universal health care is in place. Individuals with greater levels of income, many of whom have private insurance, often seek health care outside of the public sector in Brazil.

We purposively sampled ten heterosexual women, ten heterosexual men and ten men who have sex with men to allow for cross sub-group comparisons. All of the study participants had a confirmed STI diagnosis based on clinic records and discussion with attending physicians. Participants were considered heterosexual for the purposes of the study if they reported no other same sex partners in the last year. Male participants were considered MSM if they reported having at least one male sexual partner in the last year.

A physician or social worker from each clinic assisted to recruit participants by facilitating access to attending physicians who described the study to patients testing positive for STIs on a given recruitment day. If the patient expressed interest in participating, they were then provided with additional verbal and written information about the project by study staff in a private setting and signed an informed consent form indicating that they agreed to participate. None of the individuals approached declined to participate in the study. All interviews conducted were anonymous and confidential. Each participant received $US7 for their participation in the study. The study was undertaken with the ethical approval of the Committees on Human Research of the Johns Hopkins Bloomberg School of Public Health, the Oswaldo Cruz Foundation, and the Brazilian National Ethics Committee.

The in-depth interviews conducted were semi-structured in nature using a scripted field guide that focused on participants' day to day lives, relationships and sexual behavior, HIV/STI knowledge and attitudes, and STI-related care seeking. In specific we sought to understand participants' perceptions regarding their experience and treatment at the STI clinic they attended, and their STI-related knowledge and behavior change post-diagnosis. Interviews usually lasted between one and one and a half hour each and were conducted by one of two female, Brazilian interviewers trained in qualitative research methods.

To capture participants' experiences in their own words, the interviews were audiotaped with the participant's consent. The data were then transcribed verbatim in Portuguese by two study team members, including this study's first author. A list of preliminary codes was developed by the research team on the basis of the semi-structured field guides used to guide each in-depth interview. However, the list of codes was further developed and refined as interviews were conducted, transcribed, read and reviewed as well as throughout the coding process itself. Additionally, two of the study investigators most actively involved in the analysis, the first and last authors, independently coded two initial interviews from each study sub-group. Adjustments were made in the coded documents when minor differences in interpretation were identified. All transcripts were coded using the software program Atlas.ti^© ^[14]. Key domains of interest utilized in the coding process included STI-related: symptom experiences, symptom management, treatment seeking, patient-provider interactions, medication adherence, post-diagnosis health education, and post-diagnosis behavior change. Code output was compared both within and across study sub-groups.

After reading all of the coded interviews and examining the code output from each of the groups several times, a few key themes began to emerge including the role of low STI knowledge and high perceived stigma on STI-care seeking and the lack of quality health education received. We then sought to illustrate the nature of these thematic areas by developing narratives which offered a synthesis of participant's experiences and stories. Such narratives were constructed for each of the three study sub-groups. Excerpts from key passages within the coded texts were selected to help develop key points within the narratives developed for each of the study groups. These excerpts were translated into English by the first author and double-checked by the study's last author who is also bilingual in Portuguese and English.

## Results

Table 1 depicts the socio-demographic information for the thirty study participants. Most participants were in their twenties and thirties, with a median age of 32.0 (18–43) among women, 21.0 (18–32) among heterosexual men and 21.50 (18–38) among MSM. The majority of participants had low levels of formal education. The percent of participants that had some level of high school education, for example, was 40% among women, 60% among heterosexual men and 30% among MSM. Almost all of the women interviewed had a regular sexual partner (90%) and 70 percent of women reported being either married or cohabiting with their partner. Among heterosexual men, 40 percent reported having a regular partner and 30 percent reported being married. Thirty percent of MSM reported having a regular partner and one reported being married to another man. Many participants (12/30) reported having no regular income at all. Among those that did have an income the median was generally low, but varied somewhat across the three sub-groups, with a median of $US72 among women (n = 6), $US120 among heterosexual men (n = 5) and $US170 among MSM (n = 7).

**Table 1:** Basic socio-demographic characteristics of study sample participants recruited from two public STI clinics in Rio de Janeiro, Brazil (N = 30)

**Patient characteristics and experiences**	**Heterosexual women (n = 10)**	**Heterosexual men (n = 10)**	**Men who have sex with men (n = 10)**
Median age in years	32.0 (18–43)	21.0 (18–32)	21.50 (18–38)
Years of formal education			
1–8 years	60%	30%	60%
8–11 years	40%	60%	30%
12 or more	0%	10%	10%
Median monthly income	4 no income $US72 (40–72)	5 no income $US120 (24–240)	3 no income $US170 (60–360)
Married or cohabitating	70%	30%	10%
Has current regular sexual partner	90%	40%	30%
Number of sexual partners in last three months	1.0 (0–1)	1.0 (1–2)	30.0 (1–300)
Number of sexual partners in last year	1.0 (1–2)	2.0 (1–4)	50.0 (4–1500)

The median number of sexual partners also varied considerably across the three sub-groups. The median number of sex partners over the last year and during last three months was the same for heterosexual women, at 1.0, with a range of (0–1) during the last three months and (1–2) during the last year. The median number of sexual partners for heterosexual men was 2.0 (1–4) in the last year and 1.0 (1–2) in the last three months. Among MSM, the median number of sex partners during the last year was 50.0 (4–1500) and during the last three months 30.0 (1–300). One MSM reported that he had too many partners to recall during those time periods. During the course of the interviews, it became clear that several MSM interviewed exchanged sex for money on a regular basis, which in turn influenced the high numbers of sexual partners reported by those participants.

### STI-related knowledge, fears and perceived stigma prior to care-seeking

All participants delayed seeking care for at least one week after the onset of symptoms. However, there were many participants who waited much longer, such as a few months in several cases to over a year among a few cases, before seeking clinical care for their symptoms. For most participants, the main reason they sought care at an STI clinic was the existence of prolonged, "visible", "scary" and often painful symptoms including discomfort during intercourse, frequent urination, bleeding, abdominal pain, and warts or wounds around their genital organs. In interpreting the significance of these signs and cues, participants experienced considerable uncertainty. In the absence of information surrounding these symptoms, they often did not readily recognize them as being STI-related and assumed that they would resolve themselves with time as narrated below.

*"I was sick before ...but I didn't know what I had ... I got this *[the STI]*3 years ago and I didn't know exactly what was it. Anyway, the wounds eventually went away over time...but I got concerned when I saw the same wounds come back again. Then I got really worried and came here to see a doctor" *[Heterosexual male, 19 years old, with syphilis]

*"I came here [to the STI clinic] with fear, because it had been more than two months that each time I had sex I bled. At first I thought it was my period, but now I know it isn't..." *[Heterosexual female, 42 years old, with HPV]

However, there were important indications in the data that awareness and information regarding STI-related symptoms may differ across the different study sub-groups. For example, more heterosexual males and in particular the MSM interviewed, as compared to female participants, had a stronger sense that they were dealing with an STI before coming to the clinic.

Respondent: *I saw some small warts and looked for care right away*.

Interviewer: *And did you know what it was, even before your exams?*

Respondent: *Definitely, I knew when I saw it. This is the kind of disease you see the symptoms right away, just like a scratch, you can see it right away*. [Heterosexual male, 19 years old, with HPV]

*"It seems like a small wart (...) I was reading about it, it can be Condyloma Acuminatum or it can be Hemorroids." *[MSM, 19 years old, with HPV]

The MSM interviewed in the study seemed to have a more defined social network of friends and/or sexual partners within which health-related information tended to flow. While this type of network facilitated access to information about issues such as STIs, the information received did not always encourage care seeking. For example:

*"I feel a sort of pain... I mean, just after having sex, when I get relaxed... I feel a sort of pain and no one can touch it *[his penis].*If you touch it, it really hurts, I just can't... But I've talked to my friends, and they told me that this is quite normal" *[MSM, 31 years old, with gonorrhea]

Several of the study participants that suspected they had an STI prior to clinical diagnosis reported trying to alleviate their symptoms through self-medication. They reported being able to purchase medicines to treat STIs without going to the doctor by going to pharmacists that did not require prescriptions. A few participants discussed procuring alternative medicines from traditional healers. While many attempts at self-treatment were uncovered in the interviews, none of the study participants reported having gone to another clinical center or STI-related provider prior to accessing care for their current symptoms at the center where they were recruited.

The excerpts below are examples of common strategies employed by participants to self-treat and the clinical implications of such unsuccessful efforts.

*"I took an antibiotic on my own, just to see if it would disappear *[some warts]. *I was already using it for my pimples, then I decided to take a bigger dose of it" *[MSM, 19 years old, with HPV]

*"Well... Sometimes some small warts popped up on my behind... Yeah, and I bought a salve, used it, and it just disappeared. It stopped. But it's now there are some others which keep appearing." *[MSM, 19 years old, with HPV]

Again, many of the MSM participants reported receiving advice from friends regarding how to best deal with the STI-related symptoms they were experiencing.

*"Once I had a kind of anal secretion (...) But I took tetracycline on my own...Tetracycline is an antibiotic that a friend of mine told me to use, and it cured everything." *[MSM, 23, with syphilis]

For some, particularly female participants, self-medication seemed to be linked to perceived STI-related stigma and a behavioral strategy to avoid having to face this fear vis-à-vis the clinical care consultation with a physician.

*"I felt sort of abdominal pain (...) I always used some salve, you know, vaginal salve, 'cause I know those meds. I'd rather use a vaginal salve than see a doctor...I was ashamed"*. [Heterosexual female, 35 years old, with HPV]

In addition to fears or embarrassment associated with the potential of having an STI, prior experiences of perceived stigma and discrimination related to other social categories also appeared within interview discussions as a barrier to care seeking, particularly among MSM as the following excerpt reveals.

Respondent: *When I first saw it *[a wart] *I didn't pay much attention to it. I talked to a friend of mine, because he already had it, and he told me it will disappear naturally. But I got concerned, because it wasn't disappearing...*

Interviewer: *And why didn't you go to the clinic?*

Respondent: *I felt ashamed...*

Interviewer: *Why?*

Respondent: *Previously, I went to see a doctor near where I leave, and I told him that I wanted to run some exams, because I was feeling pretty bad... So he told me just like that: 'It must be AIDS, because every homosexual has it!' He was completely rude, he treated me like... Then I ran out the clinic, crying. I swore that I would never, ever go back again... *[MSM, 19 years old, with HPV]

### STI-related knowledge, fears and perceived stigma post-diagnosis

Many of the participants interviewed across all three sub-groups reported being physically examined and given a prescription, but not specifically counseled regarding the fact that they had an STI, nor the type of STI. For example:

*"The doctor just told me it was a severe illness... He didn't tell me what it was, he didn't explain anything to me. He just asked me to use this salve." *[Heterosexual female, 23 years old, with HPV]

*"He *[the physician] *just told me that I had a kind of inflammation, and that he couldn't run my exams today, that first he needed to treat my inflammation. That's all." *[Heterosexual female, 18 years old, with candidiasis]

Some participants left their consult without having received any information regarding how STIs were transmitted, as seen below.

Interviewer: *And do you have any idea how you got this STI?*

Respondent: *I just don't know. I have no clue at all...*

Interviewer: *Didn't your doctor talk with you about your STI, or about the STI risks?*

Respondent: *No, not at all*. [Heterosexual female, 42 years old, with HPV]

Interviewer: *How do you think you got this STI?*

Respondent: *Well, I really don't know. He *[the physician] *told me that I can get it by using someone else's shorts ... It could be because of that, 'cause I usually go to a friend's house, and I always use his shorts*. [Heterosexual male, 22 years old, with HPV]

Interviewer: *And how do you think you would end up with this STI?*

Respondent: *This inflammation, well I think that it's because I walk around barefoot, most of the time my stomach is wet, I am washing clothes and I get all wet and I don't warm myself up. It could be that..." *[Heterosexual female, 18 years old, candidiasis]

Perceived stigma and the lack of counseling and support received not only appeared to impact the patient's knowledge and health, but also their ability to notify their partner(s) of their STI diagnosis, as suggested in the following excerpt:

Interviewer: *Do you have any idea how you got this STI?*

Respondent: *I really don't know. This type of thing never even entered my mind before. All girls think they will never get this type of thing. People say there is a cure, but it's so embarrassing. I didn't have the courage to tell my husband about this. What would he think? He would think I was with someone else. So now I am living with a heavy conscience." *[Heterosexual female, 21 years old, with HPV]

In cases where participants did report having discussed their diagnosis with their physician, the information they received or ultimately came away with did not in most cases clear up their initial confusions and sometimes provoked additional fears and perceived stigma regarding the nature and future course of their disease.

*"A doc told me that syphilis is a blood disease, and if you leave it untreated, it becomes worse and worse...She talked to me very briefly about it, that when a disease becomes more and more severe it can even turn into another disease, it can cause other diseases. So, if this first disease becomes more severe, and if I do not achieve my cure, it can even turn into AIDS." *[Heterosexual male, 19 years old, with syphilis]

*"I could never, ever imagine this. I went to the clinic as I always do, and I got really desperate when I saw HPV in my exams. I felt really dirty, and thought that I'll have lots and lots of warts. Then I talked to my doc, and he made me even more anxious, 'cause he told me that if I do not look for treatment I could never have a child...I could never imagine that such thing will happen to someone like me." *[Heterosexual female, 22 years old, with HPV]

While the MSM interviewed generally exhibited higher levels of STI-related knowledge and in many cases prior experiences with STIs, there were almost no indications that they had any dialogue or counseling with their attending physicians regarding STI prevention. Only a minority of participants reported having discussions regarding condom use or partner notification with their attending STI care provider calling into question the quality of health education efforts at these public clinics.

## Discussion

Findings from this qualitative study suggest that STI care-seeking delays among patients attending public STI clinics in Brazil are linked to both information gaps regarding STI transmission, detection and treatment as well as the result of psychosocial constraints including perceived STI-related and other social stigma. However, the extent to which these factors may limit care-seeking behavior appears to differ by study sub-group reflecting differences in social experiences. For example, we observed that female participants appeared to possess less STI-related knowledge and greater levels of perceived STI-related stigma than male participants, resulting in additional confusion and anxiety regarding whether to seek care. MSM participants on the other hand received a considerable amount of information regarding STI from their peers, while this information and advice was not always accurate or effective. While MSM participants discussed avoiding STI-related care to avoid stigma, they appeared more concerned about being discriminated against due to their sexual orientation, rather than feeling ashamed about having an STI per se. Prior qualitative research conducted in the United States has documented the importance of social experience including race/ethnicity and gender in shaping the way in which STI-related stigma is experienced and acts as a barrier to STI-care seeking and treatment [15].

Based on these findings, additional information, education and communication efforts should be developed for the general public, specifically with regard to the early recognition of STIs and the benefits of prompt care-seeking. Such initiatives should consider tailoring their messages to specific sub-populations and take advantage of existing information channels within networks such as those among MSM. However, public health intervention efforts aimed at improving STI-related care-seeking should also address the role of STI-related stigma as well as other social stigma, such as homophobia, in order to increase their effectiveness.

The clinic encounter is a key opportunity to address patients' lack of or misinformation regarding STIs as well as perceived stigma, shame, and other fears. However, our results indicate that upon presenting at a clinic not all individuals received adequate education and prevention counseling from health care personnel. The MSM we interviewed gave almost no indication that there had been any type of dialogue regarding STI prevention during their visit. Furthermore, in some cases, participants left the clinic more anxious than when they arrived. This was predominantly due to confusion they harbored following their clinical interaction regarding the consequences of their STI diagnosis such as the relationship between STIs and HIV/AIDS or infertility. Such a lack of quality health education and counseling also represents an important missed opportunity for reducing the spread of HIV. For example, a recent study conducted among STI clinic attendees in Vitoria, Brazil documented a 6.8 percent HIV prevalence rate [16] as compared to the 0.7 overall adult prevalence rate in Brazil [17], highlighting the importance of engaging STI patients in HIV/STI prevention.

Barriers to clinicians providing post-diagnosis education and counseling may include lack of time, lack of specialized training including counseling skills or knowledge about STIs, dedicated funding for staffing, and/or some providers' understanding of their roles and responsibilities [13,18,19]. Education and counseling efforts may also be inhibited by health care workers' own beliefs and values regarding sexuality and STIs [13,19]. Negative attitudes toward female sexuality have been documented in studies of health care workers [20]. As a result, providers may be more comfortable discussing issues related to STIs with men. Based on data collected from a number of primary health care posts in Rio de Janeiro and São Paulo, Vuylsteke and colleagues (1997) found that male STI patients were more likely than females to receive information which would help in disclosing to partners as well as discuss the importance of and be given condoms [21]. Similarly, among persons attending public HIV clinics in the United States, Marks and colleagues (2002) found that a smaller percentage of MSM than both heterosexual men and women reported having had a discussion with their provider about lower sex risk during their most recent visit [19]. These authors suggest that such findings may be due to providers discomfort talking about homosexual behavior, findings similar to a recent study conducted in Brazil [22], or due to assumptions that MSM are already aware of the importance and means of prevention thus need no additional information. In turn, future studies should explore both attitudinal and structural factors that may be impeding the quality of STI education and counseling efforts offered by public health care providers, particularly across population groups with different social experiences per gender, class, sexual orientation, and/or race/ethnicity.

There are several important limitations to this study. Both the small number of participants and recruitment of a convenience sample limit the generalizations that can be made. In addition, our findings are based on persons who had ultimately presented at an STI clinic and thus do not include the perspectives of those who never seek care or only do so outside of the formal health care system. Additionally, in this study we focused on patients' perceptions alone regarding the STI quality of care. Future studies should consider eliciting both patient and provider perspectives on their communication dynamics by employing in-depth interviews with each group. The use of additional data collection methodologies including clinic observations and simulated client techniques [23] among a larger sample and across more clinics may also be useful to gain a more comprehensive picture of the quality of health education within Brazilian public STI clinics.

## Conclusion

In conclusion, the major themes that emerged from participant narratives included the importance of low STI-related knowledge and high perceived stigma, both in terms of STIs as well as other types of social stigma, on STI care-seeking delays. However, there are indications in the data that the level of STI-related knowledge and the amount and types of stigma experienced vary across the study sub-groups suggesting the need for further research on the significance and program relevance of these potential differences. Interview findings also suggest that such barriers to care seeking are not adequately addressed through ongoing health education and counseling efforts at public STI clinics and in turn critical opportunities for STI/HIV prevention are currently being missed. Our findings suggest that further research regarding the nature of ongoing barriers to the delivery of quality STI/HIV education within public STI clinics in Brazil is warranted. Information, communication and education regarding early recognition and prompt care-seeking for STIs should be developed, with consideration given to the possibility of tailoring messages tailored to specific sub-groups. To promote prompt treatment-seeking, interventions must also address both STI-specific and other forms of social stigma which may limit access to care. Efforts to further assess and respond to barriers related to the delivery of quality health education and counseling within the context of public STI clinics are also needed.

## Competing interests

The author(s) declare that they have no competing interests.

## Authors' contributions

MM was responsible for the acquisition, analysis and interpretation of data, and for the manuscript elaboration. FIB was responsible for the supervision of field activities and made substantial contributions on the manuscript elaboration, SAS made substantial contributions to the manuscript conception and elaboration, SDC participate on the analysis and interpretation of data and on the manuscript elaboration, JHP supervised all the field activities and helped the manuscript elaboration, and DK assisted in the data analysis and interpretation as well as making substantial contributions to the study conception and design and to the manuscript elaboration. All authors read and approved the final manuscript.

## Pre-publication history

The pre-publication history for this paper can be accessed here:


